# Modeling the differential effect of prescribed fire on multi-vector tick-borne tularemia disease

**DOI:** 10.1371/journal.pone.0329465

**Published:** 2025-08-11

**Authors:** Folashade B. Agusto, Joshua Atsu, Dorothy Kwarteng-Adjei, Daniel Lamptey-Mills, Samuel A. Osei

**Affiliations:** 1 University of Kansas, Lawrence, Kansas, United States of America; 2 North-West University, Potchefstroom, South Africa; 3 Kwame Nkrumah University of Science and Technology, Kumasi, Ghana; 4 University of Ghana, Accra, Ghana; 5 University of Education, Winneba, Ghana; Kerman University of Medical Sciences, IRAN, ISLAMIC REPUBLIC OF

## Abstract

Tularemia is a zoonotic disease caused by *Francisella tularensis* bacteria, a gram-negative coccobacillus-shaped bacterium. There are multiple transmission routes of the infection to humans such as consumption of contaminated food or water, handling of infected animals or bites from haematophagous arthropods (such as ticks, deer flies, or mosquitoes). In this study, we focus on transmission via the bites of ticks and developed a deterministic model of ordinary and impulsive differential equations to gain insight about the differential effect of prescribed fire on *Dermacentor variabilis* and *Amblyomma americanum* ticks and the prevalence of tularemina. We found that prescribed fire differentially reduce the number of the two ticks with *A. americanum* reduced the least compare to *D. variabilis* subsequently leading to differential increase of tularemia new infected cases in humans and rodents. Our result further indicates that the spatial arrangement of burn against unburn areas may not matter for humans and *D. variabilis* unlike *A. americanum* and the rodents which had more infected in unburn areas compare to burn areas when prescribed fire was implemented. The results of this study provide important new understandings of the intricate effect of prescribed fire on tick species, and the dynamics of the tick-borne disease.

## 1 Introduction

Tick-borne diseases pose a growing public health concern, with their incidence on the rise in various regions around the world; generally tick-borne diseases, doubled from 2006 to 2018 [1, 2]. For instance in the United States over 48,000 cases of tick-borne illnesses were reported in 2016 with close to 51,000 cases reported in 2019 [3]. These figures include reported cases from tick-borne diseases such as Lyme disease, anaplasmosis, Rocky Mountain spotted fever (RMSF), Babesiosis, ehrlichia chaffeensis ehrlichiosis, and Tularemia among others. The number of human Tularemia reported cases has been increasing in recent times in several countries from countries in North America (Canada, Mexico, United States), to several Nordic countries (Denmark, Norway, Sweden, Finland, and Iceland), and some Asian countries [4, 5]. For instance, in Europe close to 1,500 cases of Tularemia disease were reported in 2019 with over 1,000 cases reported in 2016 [6]. *Francisella tularensis* is mostly distributed in the Northern hemisphere and is not normally found in the southern hemisphere or the tropics [6].

Tularemia has recently become a significant re-emerging disease in the world. Its low infectious dose, easy, high dissemination with aerosols, and the ability to induce fatal disease make *Francisella tularensis* a potential agent of biological warfare [7–9]. This is why the Centers for Disease Control and Prevention (CDC) classified it as a category A biological weapon [4, 8].

Tularemia is a zoonotic disease caused by *Francisella tularensis* bacteria [4, 6, 10, 11], a gram-negative coccobacillus-shaped bacterium; it is also known as deer fly fever, Ohara’s fever, rabbit fever and Pahvant Valley plague [4, 12]. There are four subspecies of *Francisella tularensis* namely *tularensis* (type A), *holarctica* (type B), *novicida*, and *mediasiatica* [4, 11]; *Francisella novicida*, is a rare isolate initially isolated from a water source in Utah in 1951 [13], this is a separate species, but is often considered the fourth subspecie [13, 14]. The most virulent subspecies are *tularensis* and *holarctica* [6, 11, 15–17]. The subspecies *tularensis*, the most virulent of the two virulent subspecies, only occur in North America, while the subspecies *holarctica* is the most widespread of the subspecies, *mediasiatica* is present in central Asia, and *novicida* is the least virulent [6]. The subspecies *tularensis* and *holarctica* are the major etiological agents of tularemia in humans [4]. While the subspecies *novicida* is rarely associated with human infections [4, 18], the subspecies *mediasiatica* have never been documented in published literature to cause infection in humans [4].

Transmission of *F. tularensis* to humans occur via multiple routes, such as consumption of contaminated food or water, handling of infected animals or bites from haematophagous arthropod vectors (such as ticks, deer flies, or mosquitoes) [6, 19, 20]; other routes include contact with aquatic environment, and inhalation of aerosols [4, 21–23]. Once an individual is infected with *F. tularensis*, the incubation period ranges between 1–21 days [4, 24–28]. The symptoms of tularemia include fever, fatigue, chills, and headache. The clinical manifestation of tularemia in humans depends on the site and route of acquisition of the infection[4, 29]. There are six clinical forms of infection in humans, these include ulcero-glandular, glandular, oculo-glandular, oro-pharyngeal, pneumonic, and typhoidal forms [4, 29, 30].

Ulcero-glandular tularemia develops as a result of direct contact with an infected animal or a vector bite such as tick or deer fly; it leads to having skin lesions and lymphadenopathy. Although glandular and ulceroglandular tularemia are similar; however, glandular differs from ulceroglandular with the presence of regional lymphadenopathy with no detectable skin lesion. The oculo-glandular tularemia often develops via direct contact with contaminated water or splash of infected animal’s body fluids into the conjunctiva. Oro-pharyngeal result from ingestion of the bacterium and lead to symptoms such as pharyngitis, fever, and cervical lymphadenitis appear [31]. Pneumonic tularemia develops from the inhalation of infectious aerosols, while typhoidal tularemia develops from ingesting contaminated food and water. Moreover, pneumonic and typhoidal tularemia are considered systemic forms as they develop by the spread of bacteria through blood circulation as a systemic disease [4]. All six clinical forms of tularemia can cause secondary pleuropneumonia, meningitis, and sepsis, shock and death in infected individuals [32]. Tularemia is often diagnosed through the use of culture, serology, or molecular methods. The disease is frequently treated using quinolones, tetracyclines, or aminoglycosides. There are no licensed vaccine available for the prevention of tularemia [4, 33].

The bacterium has a wide host range including different vertebrate groups as well as invertebrates [11, 34]. However, rodents, hares, and rabbits are the principal vertebrate hosts [6], while ticks are the principal enzootic vector and reservoir and major sources of infection in human [4, 6, 35]. According to the Centers for Disease Control and Prevention (CDC), in the United States, tularemia disease can be transmitted to humans by three tick species namely *Amblyomma americanum* (lone Star tick), *Dermacentor variabilis* (american Dog tick) and *Dermacentor andersoni* (wood tick) [19]. In Europe, tularemia is transmitted by *Dermacentor reticulatus* [6]. Ticks goes through four developmental stages from eggs to larva to nymph, and then adult [36]. During each life stage, the ticks needs a blood meal to develop into the next stage of their life cycle [37].

Fire is considered a basic ecological process that keeps a variety of vegetative communities intact [38]. Prescribed fire is a common and essential land management technique in ecosystems that depend on fire, such as grasslands, open pine forests, and wetlands [39, 40]. Prescribed fire are fire set by a group of professionals under specific weather conditions to restore health to ecosystems that depend on fire [41, 42]. Prescribed fires are commonly used to control tick population in different environments by killing the ticks directly and destroying their leaf litter habitats [43]. Given the increase in recent decades of tick-borne diseases and the discovery of several new pathogens [44, 45], it is necessity to identify cost-effective and practical methods for reducing the risk of tick-borne diseases. Gleim *et al*. [43], found that extended prescribed fires not only significantly decreased tick abundance but may ultimately reduce the risk of tick-borne diseases. Furthermore, their study showed that prescribed fire also altered the composition of tick species according to the burn regimes with *Amblyomma americanum, Ixodes scapularis, and Amblyomma maculatum*, dominating *Dermacentor variabilis* in the different burn regimes [43].

Our goal in this is to investigate the differential effect of fire on a tick -borne disease via a mathematical model since prescribed fire affects tick species differently as shown by Gleim *et al*. [43]. However, fewer modeling work have been done to consider the effect of prescribed fire on tick-borne diseases [2, 46]. Guo and Agusto [2], investigated the effect of high and low intensity of prescribed fire on a single-vector tick-borne disease. The study presented a compartmental model for ticks carrying Lyme disease and incorporated the effects of prescribed fire using an impulsive system. They recommended that high-intensity prescribed burns done annually resulted in the most significant reduction in infectious nymphs, the primary carriers of Lyme disease [2]. Fulk *et al*. [46] also explored the effect of prescribed burns on the tick populations and the spread of Lyme disease by developing a spatial stage-structured tick-host model to simulate the impact of prescribed fire on tick populations. The numerical simulations explained the effects of different numbers of burns and the time between burns on tick populations. However, it was again found that consistent prescribed burning at high intensity was the most effective control method for tick populations [46]. Furthermore, Fulk and Agusto [47] showed significant increase in the number of *Ehrlichiosis* infected *A. americanum* ticks at temperature increases in the absence of prescribed burning. However, with prescribed burning, they observed a reduction in the prevalence of infectious ticks even as temperature increases to level such as 2^0^C and 4^0^C.

In this research work, we develop a compartmental model for a two-ticks two-hosts system with both tick species carrying *F. tularensis* and we investigate the differential effect of fire on the ticks and the prevalence of tularemia disease on the hosts. We are not aware of any studies considering the transmission of a single pathogen by multiple vectors. Although several studies have been done on a single-vector single-pathogen system [48–54] and co-infection of multiple pathogens in a single tick vector [55–57]. Also, some studies have considered single vector-multiple host system. For instance, Occhibove *et al*. [58] considered a single-vector, multi-host model for two tick species (*Ixodes ricinus* and *Ixodes trianguliceps*) individually infected with *Borrelia burgdorferi* and *Babesia microti*; the model further incorporated the ecological relationships with non-host species. The study aimed to understand the dynamics of tick-borne pathogens in pathosystems that differ in vector generalism. The study found that the degree of vector generalism affected pathogen transmission with different dilution outcomes. Norman *et al*. [59] also considered a two-hosts one-tick system with one host viraemic and the other not. They found that the non-viraemic hosts could either amplify the tick population leading to the persistence of the virus, or they may dilute the infection and cause it to die out.

The remainder of the work in this paper is organized as follows. In Sect 2.1, we formulate our baseline tick/tularemia disease model, compute the model basic reproduction number, and carry out a global stability analysis including sensitivity analysis to determine the parameter with the most impact on the response function (basic reproduction number). In Sect 2.3, we describe the tick model with the effect of prescribed fire using an impulsive system of ordinary differential equations and discuss prescribed fire related parameters estimated from literature. In Sect 2.4, we carry out a global sensitivity analysis of the developed models. In Sect 3, we present some simulation results, and in Sect 4 we discuss our findings and close with conclusions.

## 2 Methodology

### 2.1 Model formulation

According to the Centers for Disease Control and Prevention, tularemia disease humans become infected when they are bitten by vector bite such as ticks or deer flies or by handling infected or deceased animals, or by the consumption of contaminated food or water. In this section, we formulate a model using non-linear ordinary differential equation that accounts for the interplay between humans (a dead end host, there is no evidence of human-to-human transmission), the American dog tick (referred to as tick 1), the lone star tick (referred to as tick 2) and rodents (pathogen reservoir). This model incorporates various compartments that depict the epidemiological state of each species in the system.

In the model, the human population is divided into susceptible *S*_*H*_(*t*), exposed *E*_*H*_(*t*), asymptomatic *A*_*H*_(*t*), infected *I*_*H*_(*t*) and recovered *R*_*H*_(*t*). The total human population is denoted by *N*_*H*_(*t*) and is defined as


$$N_{H}=S_{H}+E_{H}+A_{H}+I_{H}+R_{H}.$$

The total rodent population is denoted by *N*_*M*_(*t*) and is defined as


$$N_{M}=S_{M}+I_{M}.$$

The tick population is divided by life stages, which include eggs, larvae, nymphs, and adults. Additionally, it is separated into susceptible and infected groups for larvae (*S*_*L*1_(*t*) and *I*_*L*1_(*t*)) and (*S*_*L*2_(*t*) and *I*_*L*2_(*t*)), nymphs (*S*_*N*1_(*t*) and *I*_*N*1_(*t*)) and (*S*_*N*2_(*t*) and *I*_*N*2_(*t*)), and adults (*S*_*A*1_(*t*) and *I*_*A*1_(*t*)) and *S*_*A*2_(*t*) and *I*_*A*2_(*t*) for tick 1 and tick 2, respectively. Since ticks must feed on blood to get infected, and there is no vertical transmission of the disease from parent ticks to their offspring, all tick eggs remain infection free (*S*_*E*1_(*t*) and *S*_*E*2_(*t*)).

For simplicity, we assume that human, tick and rodent populations are mixing homogeneously. The susceptible human sub-population are recruited at a rate $\pi_{H}.$ The rate at which a susceptible human progress to the exposed class is denoted by $\lambda_{H}$ and is defined as


$$\lambda_{H}=\frac{\beta_{TH1}\left(I_{L1}+I_{N1}+I_{A1}\right)+\beta_{TH2}\left(I_{L2}+I_{N2}+I_{A2}\right)}{N_{H}}.$$

This quantity $\lambda_{H}$ is the force of infection for the humans, where $\beta_{TH1}$ and $\beta_{TH2}$ are the probabilities of transmission from tick 1 to human and tick 2 to human, respectively. We have assumed that the transmission rates of the virus to humans from larvae, nymphs, and adults are the same.

The incubation period for tularemia in humans ranges 1–21 days [4, 24–28]. Furthermore, 4–19% of the infected human cases are asymptomatic [12, 60]. Thus, the exposed human subsequently becomes either asymptomatic at a rate $p\sigma_{H}$ or infected at a rate $(1\;-\;p)\sigma_{H}$ with *p* < 1. Tularemia in humans is usually treated with antibiotics such as streptomycin, gentamicin, doxycycline, and ciprofloxacin [61]. Depending on the stage of illness and the type of medication used, treatment can last as long as 10–21 days with many of the patients completely recovering from the illness [61, 62]. Hence, we assume that individuals who are infected as well as those who are asymptomatic both recover at the rate $\gamma_{H}$ according to the reciprocal of the length of the treatment days. The human population is decreased by natural death at the rate denoted by $\mu_{H}$ and by disease induced mortality denoted by $\delta_{H}$. The disease induced mortality rate for *F. tularensis tularensis* infections is about 30–35% and about 5–15% for *F. tularensis holarctica* infections when left untreated [28, 62, 63]. When treated with the appropriate antibiotics, these figure reduces to 1–3% [62, 63]. The mortality rate in patients with typhoidal tularemia is higher than those of other forms of tularemia [63]. The case fatality rate of typhoidal tularemia if untreated is approximately 35%; it is the most dangerous form of tularemia infections [63]. Untreated ulceroglandular tularemia infection has a case fatality rate of 5% [63]. Permanent immunity usually develops after a single episode of tularemia [63].

For the rodent population, the recruited at a rate into the susceptible sub-population is denoted by $\pi_{M}$. The rodents become infected following a bite from infected ticks of type 1 and 2 at the rate


$$\lambda_{M}=\frac{\beta_{TM1}\left(I_{L1}+I_{N1}+I_{A1}\right)+\beta_{TM2}\left(I_{L2}+I_{N2}+I_{A2}\right)}{N_{H}}.$$

where $\beta_{TM1}$ and $\beta_{TM2}$ are the probabilities of transmission from tick 1 to rodents and tick 2 to rodents, respectively. We have assumed that the transmission rates of the virus to rodents from larvae, nymphs, and adults are the same. Rabbits, hares, and rodents often die in large numbers during outbreaks due to their high susceptibility to the bacteria [64]. The rodent population therefore is decreased by disease induced mortality denoted by $\delta_{M}$ and by natural death at the rate by $\mu_{M}$.

For ticks, we assume that every adult tick can reproduce, regardless of whether they are susceptible or already infected, and that there is no vertical transmission of the bacteria to the eggs from infected females. The eggs develop into the larvae at rates $\tau_{E1}$ and $\tau_{E2}$, with a certain proportion dying naturally at rates $\mu_{E1}$ and $\mu_{E2}$ for tick 1 and tick 2, respectively. The susceptible larvae of both tick 1 and tick 2 become infected larvae at a rate of $\lambda_{T1}$ and $\lambda_{T2},$ respectively or remain in the susceptible larval compartment. Thus, the force of infection in tick 1 and tick 2 (or the rate at which the ticks become infected after feeding on infected rodents) is given as


$$\lambda_{T1}=\frac{\beta_{M1}I_{H}}{N_{M}}\;\text{and}\;\lambda_{T2}=\frac{\beta_{M2}I_{M}}{N_{M}},$$

where $\beta_{M1}$ and $\beta_{M2}$ represents the probabilities that infections will occur when tick 1 or tick 2 bites an infected rodents. The populations of both susceptible and infected larvae are influenced by the natural mortality rate of $\mu_{L1}$ for tick 1 and $\mu_{L2}$ for tick 2. Regardless of their infection status, both susceptible and infected larvae from both tick species mature into their respective nymph stages at the rates $\tau_{L1}$ and $\tau_{L2}.$ Following a subsequent infectious blood meal, susceptible nymphs in tick 1 and tick 2 become infected nymph at a rate of $\lambda_{T1}$ and $\lambda_{T2},$ respectively. The populations of both susceptible and infected nymphs from both tick species are reduced due to natural death rate of $\mu_{N1}$ and $\mu_{N2},$ and they mature into adults at the rate $\tau_{N1}$ and $\tau_{N2},$ respectively.

Given the assumptions above, the following nonlinear equations are given for the transmission dynamics of tularemia in two tick species:

$$\begin{array}{ccc} \frac{dS_{H}}{dt} & = & \pi_{H}-\lambda_{H}S_{H}-\mu_{H}S_{H}\\ \frac{dE_{H}}{dt} & = & \lambda_{H}S_{H}-(\sigma_{H}+\mu_{H})E_{H}\\ \frac{dA_{H}}{dt} & = & p\sigma_{H}E_{H}-(\gamma_{H}+\mu_{H})A_{H}\\ \frac{dI_{H}}{dt} & = & (1-p)\sigma_{H}E_{H}-(\gamma_{H}+\mu_{H}+\delta_{H})I_{H}\\ \frac{dR_{H}}{dt} & = & \gamma_{H}A_{H}+\gamma_{H}I_{H}-\mu_{H}R_{H}\\ \frac{dS_{M}}{dt} & = & \pi_{m}-\lambda_{M}S_{M}-\mu_{M}S_{M}\\ \frac{dI_{M}}{dt} & = & \lambda_{M}S_{M}-(\mu_{M}+\sigma_{H})I_{M}\\ \frac{dS_{E_{1}}}{dt} & = & \pi_{T1}(1-\frac{S_{E_{1}}}{K_{1}})(S_{A_{1}}+I_{A_{1}})-(\tau_{E1}+\mu_{E1})S_{E1}\\ \frac{dS_{L1}}{dt} & = & \tau_{E_{1}}S_{E1}-\lambda_{T1}S_{L1}-(\tau_{L1}+\mu_{L1})S_{L1}\\ \frac{dI_{L_{1}}}{dt} & = & \lambda_{T1}S_{L1}-(\tau_{L1}+\mu_{L1}\;)I_{L1}\\ \frac{dS_{N1}}{dt} & = & \tau_{L1}S_{L1}-\lambda_{T1}S_{N1}-(\tau_{N1}+\mu_{N1})S_{N1}\\ \frac{dI_{N1}}{dt} & = & \tau_{L1}I_{L1}+\lambda_{T1}S_{N1}-(\tau_{N1}+\mu_{N1})I_{N1}\\ \frac{dS_{A_{1}}}{dt} & = & \tau_{N1}S_{N1}-\lambda_{T1}S_{A1}-\mu_{A1}S_{A1}\\ \frac{dI_{A1}}{dt} & = & \tau_{N1}I_{N_{1}}+\lambda_{TI}S_{A1}-\mu_{A1}I_{A1}\\ \frac{dS_{E_{2}}}{dt} & = & \pi_{T2}(1-\frac{S_{E_{2}}}{K_{2}})(S_{A_{2}}+I_{A_{2}})-\left(\tau_{E2}+\mu_{E2}\right)S_{E2}\\ \frac{dS_{L2}}{dt} & = & \tau_{E2}S_{E2}-\lambda_{T2}S_{L2}-(\tau_{L2}+\mu_{L2})S_{L2}\\ \frac{dI_{L2}}{dt} & = & \lambda_{T2}S_{L2}-(\tau_{L2}+\mu_{L2})I_{L2}\\ \frac{dS_{N2}}{dt} & = & \tau_{L2}S_{L2}-\lambda_{T2}S_{N2}-(\tau_{N2}+\mu_{N2})S_{N2}\\ \frac{dI_{N2}}{dt} & = & \tau_{L2}I_{L2}+\lambda_{T2}S_{N2}-(\tau_{N2}+\mu_{N2})I_{N2}\\ \frac{dS_{A2}}{dt} & = & \tau_{N2}S_{N2}-\lambda_{T2}S_{A2}-\mu_{A2}S_{A2}\\ \frac{dI_{A2}}{dt} & = & \tau_{N2}I_{N2}+\lambda_{T2}S_{A2}-\mu_{A2}I_{A2} \end{array}\;\;\;\;\}$$ (1)

**Fig 1:**
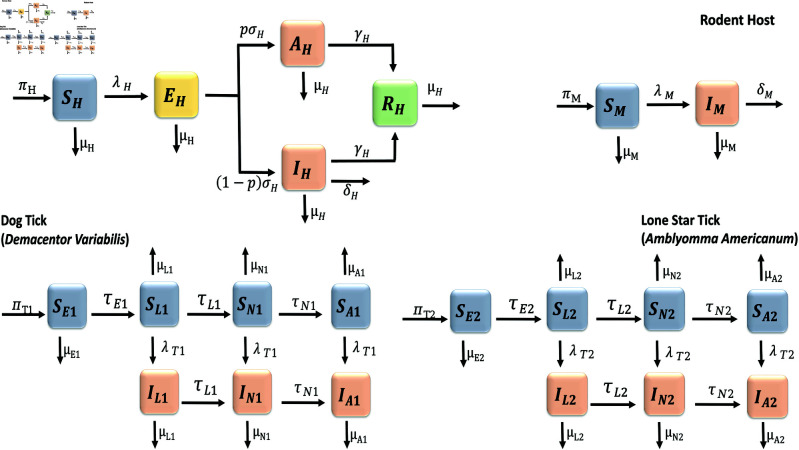
Flow diagram of the Tularemia model (1).

The flow diagram for tularemia transmission is shown in Fig 1. The corresponding parameters and variables are described in Tables 1 and 2.

**Table 1:** Description of the tularemia model (1) variables, where tick 1 represent *Dermacentor variabilis* and tick 2 represent *Amblyomma americanum.*

Variable	Description
*S*_*H*_	Number of susceptible humans
*E*_*H*_	Number of exposed humans
*A*_*H*_	Number of asymptomatic humans
*I*_*H*_	Number of infected humans
*R*_*H*_	Number of recovered humans
*S*_*M*_	Number of susceptible rodents
*I*_*M*_	Number of infected rodents
*S*_*Ei*_,*i* = 1,2	Number of susceptible eggs of ticks type i=1,2
*S*_*Li*_	Number of susceptible larvae of ticks type *i*
*I*_*Li*_	Number of infected larvae of ticks type *i*
*S*_*Ni*_	Number of susceptible nymphs of ticks type *i*
*I*_*Ni*_	Number of infected nymphs of ticks type *i*
*S*_*Ai*_	Number of susceptible adults of ticks type *i*
*I*_*Ai*_	Number of infected adults of ticks type *i*

**Table 2:** Description and values of the tularemia model (1) parameters, where tick 1 represent *Dermacentor variabilis* and tick 2 represent *Amblyomma americanum*. The morality rates for *D. variabilis* (tick 1) is calculated as “mortality rate = 1–survival rate" [70]. The survival rates for *D. variabilis* is given in [68]; the average of these survival rates for the different life stages are determined and the mortality rate is then computed.

Parameter	Description	Value	Reference
$\pi_{H}$	Human birth rate	0.011	[65]
$\beta_{TH1}$	Tick 1-to-human transmission probability	0.2	[66]
$\beta_{TH2}$	Tick 2-to-human transmission probability	0.1	[46]
$\sigma_{H}$	Disease progression rate	1/21–1	[24–28]
p	Proportion infectious	0.04—0.19	[12, 60]
$\gamma_{H}$	Human recovery rate	1/21–1/10	[61, 62]
$\mu_{H}$	Human natural death rate	0.0104	[67]
$\delta_{H}$	Human disease-induced death rate	0.03–0.3	[28, 62, 63]
$\pi_{M}$	Rodent birth rate	0.02	[55]
$\beta_{TM1}$	Rodent-to-tick 1 transmission probability	0.2	[66]
$\beta_{TM2}$	Rodent-to-tick 2 transmission probability	0.1	[46]
$\mu_{M}$	Rodent death rate	0.01	[55]
$\delta_{M}$	Rodent disease-induced death rate	0.01	[55]
$\pi_{T1}$	Birth rate of tick 1 (*Dermacentor variabilis*)	4,500	[68]
*K*_1_	Carrying capacity for tick 1	10,000	assumed
$\beta_{M1}$	Tick 1-to-rodent transmission probability	0.2	[66]
$\tau_{E1}$	Maturation rate of egg to larva for tick 1	0.2	assumed
$\tau_{L1}$	Maturation rate of larva to nymph for tick 1	0.2	assumed
$\tau_{N1}$	Maturation rate of nymph to adult for tick 1	0.2	assumed
$\mu_{E1}$	Eggs in-viability rate of tick 1	0.0174	[68]
$\mu_{L1}$	Mortality rate of the larvae of tick 1	0.0294	[68]
$\mu_{N1}$	Mortality rate of the nymph of tick 1	0.0296	[68]
$\mu_{A1}$	Mortality rate of the adult of tick 1	0.0137	[68]
$\pi_{T2}$	Birth rate of tick 2 (*Amblyomma americanum*)	6,000	[47, 69]
*K*_2_	Carrying capacity for tick 2	120,000	assumed
$\beta_{M2}$	Tick 2-to-rodent transmission probability	0.1	[46]
$\tau_{E2}$	Maturation rate of egg to larva for tick 2	0.2	[47, 69]
$\tau_{L2}$	Maturation rate of larva to nymph for tick 2	0.2	[47, 69]
$\tau_{N2}$	Maturation rate of nymph to adult for tick 2	0.2	[47, 69]
$\mu_{E2}$	Eggs in-viability rate of tick 2	0.008	[47, 69]
$\mu_{L2}$	Mortality rate of the larvae of tick 2	0.005	[47, 69]
$\mu_{N2}$	Mortality rate of the nymph of tick 2	0.004	[47, 69]
$\mu_{A2}$	Mortality rate of the adult of tick 2	0.003	[47, 69]

### 2.2 Analysis of the model

#### 2.2.1 Basic qualitative properties.

#### Positivity and boundedness of solutions.

For the tularemia model (1) to be epidemiologically meaningful, it is important to prove that all its state variables are non-negative for all time. In other words, solutions of the model system (1) with non-negative initial data will remain non-negative for all time *t* > 0.

**Lemma 1.** Let the initial data F(0)≥0 , where $F(t)=(S_{H}(t),E_{H}(t),A_{H}(t),I_{H}(t),R_{H}(t),$ $S_{M}(t),I_{M}(t),S_{Ei}(t),S_{Li}(t),I_{Li}(t),S_{Ni}(t),I_{Ni}(t),S_{Ai}(t),I_{Ai}(t))$, where i=1,2. Then the solutions *F*(*t*) of the tularemia model (1) are non-negative for all *t* > 0. Furthermore


$${lim sup}_{t\to \infty }N_{H}(t)\leq \frac{\pi_{H}}{\mu_{H}},\;\;{lim sup}_{t\to \infty }N_{M}(t)\leq \frac{\pi_{M}}{\mu_{M}},\;\;\text{ and }\;\;{lim sup}_{t\to \infty }N_{Ti}(t)\leq \frac{\pi_{Ti}}{\mu_{Ti}}.$$

where


$$N_{H}(t)=S_{H}(t),E_{H}(t),A_{H}(t),I_{H}(t),R_{H}(t),\;\;\;\;N_{M}(t)=S_{M}(t),I_{M}(t),$$

and


$$N_{Ti}(t)=S_{Ei}(t),S_{Li}(t),I_{Li}(t),S_{Ni}(t),I_{Ni}(t),S_{Ai}(t),I_{Ai}(t).$$

The proof of Lemma 1 is given in S1 Appendix.

#### Invariant regions.

The tularemia model (1) will be analyzed in a biologically-feasible region as follows. Consider the feasible region


$$\Omega =\Omega_{H}\cup \Omega_{M}\cup \Omega_{T1}\cup \Omega_{T2}\subset {\mathbb{R}}_{+}^{5}\times {\mathbb{R}}_{+}^{2}\times {\mathbb{R}}_{+}^{7}\times {\mathbb{R}}_{+}^{7},$$

with,


$$\Omega_{H}=\{(S_{H}(t),E_{H}(t),A_{H}(t),I_{H}(t),R_{H}(t))\in {\mathbb{R}}_{+}^{5}\;:\;N_{H}(t)\leq \frac{\pi_{H}}{\mu_{H}}\},$$

$$\Omega_{M}=\{(S_{M}(t),I_{M}(t))\in {\mathbb{R}}_{+}^{2}\;:\;N_{M}(t)\leq \frac{\pi_{M}}{\mu_{M}}\},$$

and


$$\Omega_{Ti}=\{(S_{Ei}(t),S_{Li}(t),I_{Li}(t),S_{Ni}(t),I_{Ni}(t),S_{Ai}(t),I_{Ai}(t))\in {\mathbb{R}}_{+}^{7}\;:\;N_{Ti}(t)\leq \frac{\pi_{Ti}}{\mu_{Ti}}\}.$$

**Lemma 2.** The region $\Omega =\Omega_{H}\cup \Omega_{M}\cup \Omega_{T1}\cup \Omega_{T2}\subset {\mathbb{R}}_{+}^{5}\times {\mathbb{R}}_{+}^{2}\times {\mathbb{R}}_{+}^{7}\times {\mathbb{R}}_{+}^{7}$ is positively-invariant for the model (1) with non-negative initial conditions in ${\mathbb{R}}_{+}^{21}$.

The prove of Lemma 2 is given in S2 Appendix. In the next section, the conditions for the existence and stability of the equilibria of the model are stated.

#### 2.2.2 Stability of disease-free equilibrium (DFE).

The tularemia model has a disease-free equilibrium (DFE). The DFE is obtained by setting the right-hand sides and infected variables of the Eq (1) to zero. The system has four equilibria ${ℰ}_{0}=({ℰ}_{0}^{1},\;{ℰ}_{0}^{2},\;{ℰ}_{0}^{3},\;{ℰ}_{0}^{4})$. The equilibrium ${ℰ}_{0}^{1}$, involves humans and rodents only; while ${ℰ}_{0}^{2}$ involves, humans, rodents and *D. variabilis* only; the equilirium ${ℰ}_{0}^{3}$ involves humans, rodents and *A. americanum* only. Lastly, the ${ℰ}_{0}^{4}$ involves humans, rodents and the two tick species. The expression for these equiliria are given as


$${ℰ}_{0}^{1}=(S_{H}^{*},\;S_{M}^{*},\;0,\;0,\;0,\;0,\;0,\;0,\;0,\;0)$$

$$=(\frac{\pi_{H}}{\mu_{H}},\;\frac{\pi_{M}}{\mu_{M}},\;0,\;0,\;0,\;0,\;0,\;0,\;0,\;0),$$

$${ℰ}_{0}^{2}=(S_{H}^{*},\;S_{M}^{*},\;S_{E1}^{*},S_{L1}^{*},S_{N1}^{*},S_{A1}^{*},\;0,\;0,\;0,\;0)$$

$$S_{H}^{*}=\frac{\pi_{H}}{\mu_{H}},\;\;S_{M}^{*}\;=\;\frac{\pi_{M}}{\mu_{M}},$$

$$S_{E1}^{*}=\frac{K_{1}(\pi_{T1}\tau_{E1}\tau_{L1}\tau_{N1}-n_{1}n_{2}n_{3}\mu_{A1})}{\tau_{N1}\tau_{L1}\tau_{E1}\pi_{T1}},\;\;S_{L1}^{*}\;=\;\frac{K_{1}(\pi_{T1}\tau_{E1}\tau_{L1}\tau_{N1}-n_{1}n_{2}n_{3}\mu_{A1})}{n_{2}\tau_{N1}\tau_{L1}\pi_{T1}},$$

$$S_{N1}^{*}=\frac{K_{1}(\pi_{T1}\tau_{E1}\tau_{L1}\tau_{N1}-n_{1}n_{2}n_{3}\mu_{A1})}{n_{2}n_{3}\pi_{T1}\tau_{N1}},\;\;S_{A1}^{*}\;=\;\frac{K_{1}(\pi_{T1}\tau_{E1}\tau_{L1}\tau_{N1}-n_{1}n_{2}n_{3}\mu_{A1})}{n_{2}n_{3}\pi_{T1}\mu_{A1}},$$

$${ℰ}_{0}^{3}=(S_{H}^{*},\;S_{M}^{*},\;0,\;0,\;0,\;0,\;S_{E2}^{*},S_{L2}^{*},S_{N2}^{*},S_{A2}^{*})$$

$$S_{H}^{*}=\frac{\pi_{H}}{\mu_{H}},\;\;S_{M}^{*}\;=\;\frac{\pi_{M}}{\mu_{M}},$$

$$S_{E2}^{*}=\frac{K_{2}(\pi_{T2}\tau_{E2}\tau_{L2}\tau_{N2}-n_{4}n_{5}n_{6}\mu_{A2})}{\tau_{N2}\tau_{L2}\tau_{E2}\pi_{T2}},\;\;S_{L2}^{*}\;=\;\frac{K_{2}(\pi_{T2}\tau_{E2}\tau_{L2}\tau_{N2}-n_{4}n_{5}n_{6}\mu_{A2})}{n_{5}\tau_{N2}\tau_{L2}\pi_{T2}},$$

$$S_{N2}^{*}=\frac{K_{2}(\pi_{T2}\tau_{E2}\tau_{L2}\tau_{N2}-n_{4}n_{5}n_{6}\mu_{A2})}{n_{5}n_{6}\pi_{T2}\tau_{N2}},\;\;S_{A2}^{*}\;=\;\frac{K_{2}(\pi_{T2}\tau_{E2}\tau_{L2}\tau_{N2}-n_{4}n_{5}n_{6}\mu_{A2})}{n_{5}n_{6}\pi_{T2}\mu_{A2}},$$

$${ℰ}_{0}^{4}=(S_{H}^{*},\;S_{M}^{*},S_{E1}^{*},S_{L1}^{*},S_{N1}^{*},S_{A1}^{*},\;S_{E2}^{*},S_{L2}^{*},S_{N2}^{*},S_{A2}^{*})$$

$$S_{H}^{*}=\frac{\pi_{H}}{\mu_{H}},\;\;S_{M}^{*}\;=\;\frac{\pi_{M}}{\mu_{M}},$$

$$S_{E1}^{*}=\frac{K_{1}(\pi_{T1}\tau_{E1}\tau_{L1}\tau_{N1}-n_{1}n_{2}n_{3}\mu_{A1})}{\tau_{N1}\tau_{L1}\tau_{E1}\pi_{T1}},\;\;S_{L1}^{*}\;=\;\frac{K_{1}(\pi_{T1}\tau_{E1}\tau_{L1}\tau_{N1}-n_{1}n_{2}n_{3}\mu_{A1})}{n_{2}\tau_{N1}\tau_{L1}\pi_{T1}}$$

$$S_{N1}^{*}=\frac{K_{1}(\pi_{T1}\tau_{E1}\tau_{L1}\tau_{N1}-n_{1}n_{2}n_{3}\mu_{A1})}{n_{2}n_{3}\pi_{T1}\tau_{N1}},\;\;S_{A1}^{*}\;=\;\frac{K_{1}(\pi_{T1}\tau {\;}_{E}1\tau_{L1}\tau_{N1}-n_{1}n_{2}n_{3}\mu_{A1})}{n_{2}n_{3}\pi_{T1}\mu_{A1}}$$

$$S_{E2}^{*}=\frac{K_{2}(\pi_{T2}\tau_{E2}\tau_{L2}\tau_{N2}-n_{4}n_{5}n_{6}\mu_{A2})}{\tau_{N2}\tau_{L2}\tau_{E2}\pi_{T2}},\;\;S_{L2}^{*}\;=\;\frac{K_{2}(\pi_{T2}\tau_{E2}\tau_{L2}\tau_{N2}-n_{4}n_{5}n_{6}\mu_{A2})}{n_{5}\tau_{N2}\tau_{L2}\pi_{T}2}$$

$$S_{N2}^{*}=\frac{K_{2}(\pi_{T2}\tau_{E2}\tau_{L2}\tau_{N2}-n_{4}n_{5}n_{6}\mu_{A2})}{n_{5}n_{6}\pi_{T2}\tau_{N2}},\;\;S_{A2}^{*}\;=\;\frac{K_{2}(\pi_{T2}\tau_{E2}\tau_{L2}\tau_{N2}-n_{4}n_{5}n_{6}\mu_{A2})}{n_{5}n_{6}\pi_{T2}\mu_{A2}},$$

where $n_{1}=\tau_{E1}+\mu_{E1}),\;n_{2}=\tau_{L1}+\mu_{L1}),\;n_{3}=\tau_{N1}+\mu_{N1},\;n_{4}=\tau_{E2}+\mu_{E2},\;n_{5}=\tau_{L2}+\mu_{L2},\;n_{6}=\tau_{N2}+\mu_{N2}.$ We will focus on the the stability of ${ℰ}_{0}^{4}$, the stability of the other equiliria ${ℰ}_{0}=({ℰ}_{0}^{1},\;{ℰ}_{0}^{2},\;{ℰ}_{0}^{3})$ can be determined using the same approach. Thus, the stability of ${ℰ}_{0}^{4}$ can be established using the next generation operator method on system (1). Taking $E_{H},\;A_{H},\;I_{H},\;R_{H},\;I_{M},\;I_{L1},$ $I_{N1},I_{A1},I_{L2},I_{N2}$, and *I*_*A*2_ as the infected compartments and then using the aforementioned notation, the Jacobian *F* and *V* matrices for new infectious terms and the remaining transfer terms, respectively, are defined as:


$$F=(\begin{array}{cccccccccc} 0 & 0 & 0 & 0 & \beta_{TH1} & \beta_{TH1} & \beta_{TH1} & \beta_{TH2} & \beta_{TH2} & \beta_{TH2}\\ 0 & 0 & 0 & 0 & 0 & 0 & 0 & 0 & 0 & 0\\ 0 & 0 & 0 & 0 & 0 & 0 & 0 & 0 & 0 & 0\\ 0 & 0 & 0 & 0 & \beta_{TM1} & \beta_{TM1} & \beta_{TM1} & \beta_{TM2} & \beta_{TM2} & \beta_{TM2}\\ 0 & 0 & 0 & \frac{\beta_{M1}S_{L1}^{*}}{S_{M}^{*}} & 0 & 0 & 0 & 0 & 0 & 0\\ 0 & 0 & 0 & \frac{\beta_{M1}S_{N1}^{*}}{S_{M}^{*}} & 0 & 0 & 0 & 0 & 0 & 0\\ 0 & 0 & 0 & \frac{\beta_{M1}S_{A1}^{*}}{S_{M}^{*}} & 0 & 0 & 0 & 0 & 0 & 0\\ 0 & 0 & 0 & \frac{\beta_{M2}S_{L2}^{*}}{S_{M}^{*}} & 0 & 0 & 0 & 0 & 0 & 0\\ 0 & 0 & 0 & \frac{\beta_{M2}S_{N2}^{*}}{S_{M}^{*}} & 0 & 0 & 0 & 0 & 0 & 0\\ 0 & 0 & 0 & \frac{\beta_{M2}S_{A2}^{*}}{S_{M}^{*}} & 0 & 0 & 0 & 0 & 0 & 0 \end{array})$$

$$V=(\begin{array}{cccccccccc} k_{1} & 0 & 0 & 0 & 0 & 0 & 0 & 0 & 0 & 0\\ -(1-p)\sigma_{H} & k2 & 0 & 0 & 0 & 0 & 0 & 0 & 0 & 0\\ -p\sigma_{H} & 0 & k_{3} & 0 & 0 & 0 & 0 & 0 & 0 & 0\\ 0 & 0 & 0 & k_{4} & 0 & 0 & 0 & 0 & 0 & 0\\ 0 & 0 & 0 & 0 & k_{5} & 0 & 0 & 0 & 0 & 0\\ 0 & 0 & 0 & 0 & -\tau_{L1} & k_{6} & 0 & 0 & 0 & 0\\ 0 & 0 & 0 & 0 & 0 & -\tau_{N1} & \mu_{A1} & 0 & 0 & 0\\ 0 & 0 & 0 & 0 & 0 & 0 & 0 & k_{7} & 0 & 0\\ 0 & 0 & 0 & 0 & 0 & 0 & 0 & -\tau_{L2} & k_{8} & 0\\ 0 & 0 & 0 & 0 & 0 & 0 & 0 & 0 & -\tau_{N2} & \mu_{A2} \end{array})$$

where, $k_{1}=\sigma_{H}+\mu_{H},\;k_{2}=\gamma_{H}+\mu_{H},\;k_{3}=\gamma_{H}+\mu_{H}+\delta_{H},\;k_{4}=\mu_{M}]+\delta_{M}],\;k_{5}=\tau_{L1}+\mu_{L1},$ $\;k_{6}=\tau_{N1}+\mu_{N1},\;k_{7}=\tau_{L2}+\mu_{L2},\;k_{8}=\tau_{N2}+\mu_{N2}$. Therefore, using the definition of the reproduction number ${ℛ}_{0}$ from [71] we have


$${ℛ}_{0}=\rho (FV^{-1})=\sqrt{{ℛ}_{M1}{ℛ}_{T1}+{ℛ}_{M2}{ℛ}_{T2}}$$

where *ρ* is the spectral radius and


$${ℛ}_{M1}=\frac{\beta_{TM1}}{k_{4}},\;\;\;\;{ℛ}_{M2}\;=\;\frac{\beta_{TM2}}{k_{4}},$$

$${ℛ}_{T1}=\frac{\beta_{M1}[S_{L1}^{*}(k_{6}\mu_{A1}+\mu_{A1}\tau_{L1}+\tau_{L1}\tau_{N1})+S_{N1}^{*}k_{5}(\mu_{A1}+\tau_{N1})+S_{A1}^{*}k_{5}k_{6}]}{S_{M}^{*}k_{5}k_{6}\mu_{A1}}$$

$${ℛ}_{T2}=\frac{\beta_{M2}[S_{L2}^{*}(k_{8}\mu_{A2}+\mu_{A2}\tau_{L2}+\tau_{L2}\tau_{N2})+S_{N2}^{*}k_{7}(\mu_{A2}+\tau_{N2})+S_{A2}^{*}k_{7}k_{8}]}{S_{M}^{*}k_{7}k_{8}\mu_{A2}}$$

The expressions ${ℛ}_{M1}$ and ${ℛ}_{M2}$ are the average number of secondary infections in rodents due to infectious *D. variabilis* and *A. americanum* while ${ℛ}_{T1}$ and ${ℛ}_{T2}$ are the average number of infections in ticks 1 and 2 due to an infectious rodents. Furthermore, using Theorem 2 in [71], the following result is established.

**Lemma 3.** The disease-free equilibrium (DFE) of the tularemia model (1) is locally asymptotically stable (LAS) if ${ℛ}_{0}<1$ and unstable if ${ℛ}_{0}>1$.

The quantity ${ℛ}_{0}$ is basic reproduction number and it is defined as the average number of new infections that result from one infectious individual in a population that is fully susceptible. [71–74]. The epidemiological significance of Lemma 3 is that tularemia model (1) will be eliminated from within a herd if the reproduction number ($R_{0}$) can be brought to (and maintained at) a value less than unity.

### 2.3 Tick model with prescribed fire

In this section, we consider the effect of fire on ticks and the rodent population, and the subsequent effect on the incidence and prevalence of the disease on humans. Note that fire is not explicitly incorporated into the tularemia model (1); rather, we consider the effect of fire on population size after the burns. To introduce the effect of prescribed fire into the tularemia disease model we have the following system of non-autonomous impulsive differential equations.

$$\begin{array}{ccc} \frac{dS_{H}}{dt} & = & \pi_{H}-\lambda_{H}S_{H}-\mu_{H}S_{H}\\ \frac{dE_{H}}{dt} & = & \lambda_{H}S_{H}-(\sigma_{H}+\mu_{H})E_{H}\\ \frac{dA_{H}}{dt} & = & p\sigma_{H}E_{H}-(\gamma_{H}+\mu_{H})A_{H}\\ \frac{dI_{H}}{dt} & = & (1-p)\sigma_{H}E_{H}-(\gamma_{H}+\mu_{H}+\delta_{H})I_{H}\\ \frac{dR_{H}}{dt} & = & \gamma_{H}A_{H}+\gamma_{H}I_{H}-\mu_{H}R_{H}\\ \frac{dS_{M}}{dt} & = & \pi_{M}-\lambda_{M}S_{M}-\mu_{M}S_{M}\\ \frac{dE_{M}}{dt} & = & \lambda_{M}S_{M}-(\mu_{M}+\sigma_{H})I_{M}\\ \frac{dS_{E_{i}}}{dt} & = & \pi_{Ti}(1-\frac{S_{E_{i}}}{K_{i}})(S_{A_{i}}+I_{A_{i}})-(\tau_{Ei}+\mu_{Ei})S_{Ei}\\ \frac{dS_{Li}}{dt} & = & \tau_{E_{i}}S_{Ei}-\lambda_{Ti}S_{Li}-(\tau_{Li}+\mu_{Li})S_{Li}\\ \frac{dI_{L_{i}}}{dt} & = & \lambda_{Ti}S_{Li}-(\tau_{Li}+\mu_{Li}\;)I_{Li}\\ \frac{dS_{Ni}}{dt} & = & \tau_{Li}S_{Li}-\lambda_{Ti}S_{Ni}-(\tau_{Ni}+\mu_{Ni})S_{Ni}\\ \frac{dI_{Ni}}{dt} & = & \tau_{Li}I_{Li}+\lambda_{Ti}S_{Ni}-(\tau_{Ni}+\mu_{Ni})I_{Ni}\\ \frac{dS_{A_{i}}}{dt} & = & \tau_{Ni}S_{Ni}-\lambda_{Ti}S_{Ai}-\mu_{Ai}S_{Ai}\\ \frac{dI_{Ai}}{dt} & = & \tau_{Ni}I_{N_{i}}+\lambda_{Ti}S_{Ai}-\mu_{Ai}I_{Ai} \end{array}\;\;\;\;\}\;\;t_{n}\neq nT,\;n\in \mathbb{N}$$ (2)

subject to the prescribed fire impulsive condition

$$\begin{array}{c} S_{H}(nT^{+})\;=S_{H}(nT^{-}),\;E_{H}(nT^{+})\;=\;E_{H}(nT^{-}),\;A_{H}(nT^{+})\;=A_{H}(nT^{-}),\\ I_{H}(nT^{+})\;=\;I_{H}(nT^{-}),\;R_{H}(nT^{+})\;=R_{H}(nT^{-}),\\ S_{M}(nT^{+})\;=(1-\nu_{M})S_{M}(nT^{-}),\;\;\;I_{M}(nT^{+})\;=\;(1-\nu_{M})l_{M}(nT^{-}),\\ S_{Ei}(nT^{+})\;=\;(1-\nu_{Ei})S_{Ei}(nT^{-})\\ S_{Li}(nT^{+})\;=\;(1-\nu_{Li})S_{Li}(nT^{-}),\;\;\;I_{Li}(nT^{+})\;=\;(1-\nu_{Li})S_{Li}(nT^{-}),\\ S_{Ni}(nT^{+})\;=\;(1-\nu_{Ni})S_{Ni}(nT^{-}),\;\;I_{Ni}(nT^{+})\;=\;(1-\nu_{Ni})I_{Ni}(nT^{-}),\\ S_{Ai}(nT^{+})\;=\;(1-\nu_{Ai})S_{Ai}(nT^{-}),\;\;\;I_{Ai}(nT^{+})\;=\;(1-\nu_{Ai})I_{Ai}(nT^{-}), \end{array}\}\;\;t_{n}=nT,\;n\in \mathbb{N},$$ (3)

where *t*_*n*_ is the times that prescribed fire is implemented, which may be fixed or non-fixed; in this study we will consider the case with fixed times. The parameters $\nu_{j}$, where j=Ei,Li,Ni,Ai,M are the proportion of tick type i=1,2 and mice population that is reduced by the fire. In Sect 2.3.1 below, we discuss how these parameters are estimated using data from literature.

#### 2.3.1 Estimating the reduction proportion $\nu_{j}$ due to prescribed fire.

To estimate the proportion ($\nu_{Ei}$, $\nu_{Li}$, $\nu_{Ni}$, $\nu_{Ai},\;\;i=1.2$, and $\nu_{M}$) reduced in the ticks and rodent populations due to fire, we used data from [75, 76]. The study sites in [75] were located in Western Illinois University’s Alice L. Kibbe Field Station located in Warsaw, Hancock County, Illinois, USA. These sites consisted of several woodlands (oak-hickory, oak barrens), floodplain forests, restored tallgrass prairies and hill prairies. Low intensity burns were carried out at these sites; for low intensity burns, the heights of most flame were less than 1 meter and plant burnt were limited to the understory vegetation [77]. The last time the entire study site was burnt was in 2004 (B04) and two additional burns were later carried out in spring of 2014 (B14) and 2015 (B15).

Following the burns, ticks were collected by sweeping through the vegetation with a $1{\text{m}}^{2}$ flannel cloth attached to a bamboo stick; this is known as flagging method. This was repeated every two weeks when the vegetation was dry between 12:00 and 18:00 hours from 9 May 2015 to 30 October 2015 and 22 April 2016 to 4 November 2016. To ensure tick mortality, the flannel cloth was placed in a sealed bag and frozen for three days. The ticks were later removed and identified using taxonomic keys, and their DNA extracted for pathogen testing. A total of 23 *D. variabilis*, 54 *Ixodes scapularis*, and 2788 *A. americanum* ticks were collected during the two consecutive years 2015 and 2016. A total of 23 *D. variabilis* were collected, *n* = 17 (74%) were adults, *n* = 2 (9%) were nymphs, and *n* = 4 (17%) were larvae. For *I. scapularis*, 4% (*n* = 2) of the 54 collected, were adults, 22% (*n* = 12) were nymphs, and 74% (n = 40) were larvae. In the case of *A. americanum*, the ticks collected in B04 made up 51% of the collection (*n* = 1433), while those collected in B14 made up 37% (*n* = 1045) of the collections, and those collected in B15 constituted 11% (n=307) of the collection. Of these ticks, 2% (*n* = 67) were adults, 4% (*n* = 107) were nymphs, and 93% (*n* = 2614) were larvae. Although *I. scapularis* were collected, we only estimate the parameters quantifying the reduction in the tick population due to fire using the data for *A. americanum* and *D. variabilis* since they are competent vector for the tuleramia.

Note that the study did not indicate if these were data for the pre-burn or post burn number of *I. scapularis* collected, nor did it provide data for the number of mice that were caught. So, we use the data for the rodent population provided in [76]. This study have data for six different rodent populations namely California kangaroo rat (*Dipodomys californicus californicus*), brush mouse (*Peromyscus boylii*), pin$\tilde{o}$n mouse (*P. truei sequoiensis*), deer mouse (*P. maniculatus gambelii*), dusky-footed woodrat (*Neotoma fuscipes*), and western harvest mouse (*Reithrodontomys megalotis longicaudus*). After the fire treatment, about half as many rodents were trapped at the treated sites compared with control sites. At the two study sites (MG and DBP) only six tick species were found namely, *Ixodes pacificus, Ixodes jellisoni, Ixodes spinipalpis, Ixodes woodi, Dermacentor occidentalis* and *Dermacentor parumapertus*. The data for the *Dermacentor* subspecies (*occidentalis* and *parumapertus*) were not use in estimating the proportions since we are not aware of the transmission efficiency of these vectors unlike *Dermacentor andersoni* [19] which we do not have data for.

**Estimating parameters $\nu_{Li},\;\nu_{Ni},\;\nu_{Ai},\;\nu_{M},\;\;i=1,2$:** To estimate these parameters, we divide the numbers collected in each age group by the total number collected and subtract the proportion obtained from 1 to obtain the proportion reduced by fire. For instance, for *D. variabilis*, we have $\nu_{A1}\;=\;1\;-\;17/23\;=\;0.2609$ for the adult population. For nymphs it is $\nu_{N1}\;=\;1\;-\;2/23\;=\;0.9130$, and $\nu_{L1}\;=\;1-4/23\;=\;0.8261$ for larvae. The proportion for *A. americanum* are similarly estimated as $\nu_{A1}\;=\;1\;-\;67/2788$, $\nu_{N1}=1-107/2788$, and $\nu_{L1}=1-2614/2788$. Lastly, the proportion for the rodent population using the data in [76] is given as $\nu_{M}=1-17/23$.

### 2.4 Global sensitivity analysis

Sensitivity analysis procedure is often used to determine the impact and contribution of the model parameters to model outputs (such as the infected population) [78–80]. Results of the sensitivity analysis help to identify the best parameters to target for intervention or for future surveillance data gathering. We implement a global sensitivity analysis using Latin Hypercube Sampling (LHS) and partial rank correlation coefficients (PRCC) to assess the impact of parameter uncertainty and the sensitivity of these key model outputs. The LHS method is a stratified sampling technique without replacement this allows for an efficient analysis of parameter variations across simultaneous uncertainty ranges in each parameter [81–84]. The PRCC on the other hand measures the strength of the relationship between the parameters and the model outcome, stating the degree of the impact each parameter has on the model outcome [81–84].

We start by generating the LHS matrices and assuming all the model parameters are uniformly distributed except for the parameters representing number of burns (pulses) and the time between burns (*τ*). With these parameters we sampled their values from two pseudo-Poisson distributions that exclude zero as in [46, 47]. For instance, Fulk *et al*. [46, 47], sampled the values for pulses from a Poisson distribution centered on 10 (since they ran a scenario for 10 years) while *τ* was sampled from a Poisson distribution centered on 10. Once the initial distributions are created, “any zeros in either sample were changed to ones to avoid issues with implementing the burns" [46, 47]. We then carry out a total of 1,000 simulations (runs) of the model for the LHS matrix, using the parameter values given in Table 2; the minimums and maximums for each parameter is set to ±20% of the baseline values respectively and the reproduction number (${ℛ}_{0}$) as the response function for tularemia model (1). We also considered other response functions at the end of the simulation for the tularemia models (1) and (2); these are the infected humans (*I*_*H*_), the infected rodents (*I*_*M*_), the sum of infected *D. variabilis* ticks in all life stages ($I_{L1}+I_{N1}+I_{A1}$), and the sum of infected *A. americanum* ticks in all life stages ($I_{L2}+I_{N2}+I_{A2}$).

#### 2.4.1 Global sensitivity analysis for tularaemia model (1).

**Fig 2:**
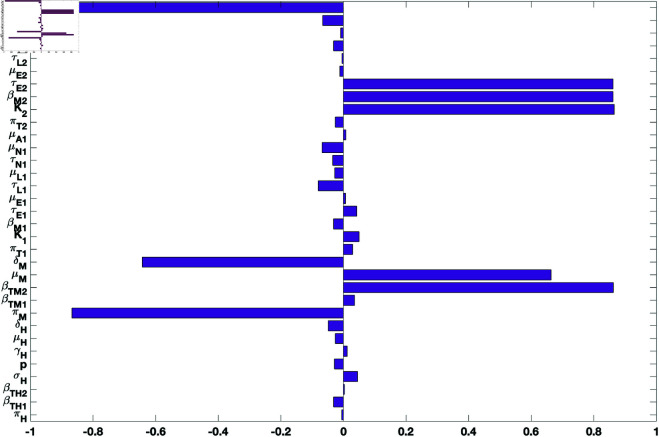
PRCC values for the tularaemia model (1), using the reproduction number ${ℛ}_{0}$ as response functions and parameter values in Table 2 with ranges that are ±20% from the baseline values.

The outcome of the global sensitivity analysis for tularaemia model (1) using the reproduction number (${ℛ}_{0}$) is shown in Fig 2. The parameters with significant effect on the reproduction number are those parameters whose sensitivity index have significant p-values less than or equal to 0.05. The parameters with the most impacts on (${ℛ}_{0}$), are rodent birth rate ($\pi_{M}$), rodent transmission probability ($\beta_{TM2}$) to tick 2 (*A. americanum*), rodent death ($\mu_{M}$) and disease induced mortality ($\delta_{M}$) rate, *A. americanum* carrying capacity (*K*_2_), transmission probability ($\beta_{M2}$) of tularemia to rodents from *A. americanum*, and the maturation rate ($\tau_{E2}$) of *A. americanum* larvae and its adult death rate ($\mu_{A2})$. Notice that the parameters with significant effects on ${ℛ}_{0}$ are related to the rodents and *A. americanum*.

**Fig 3:**
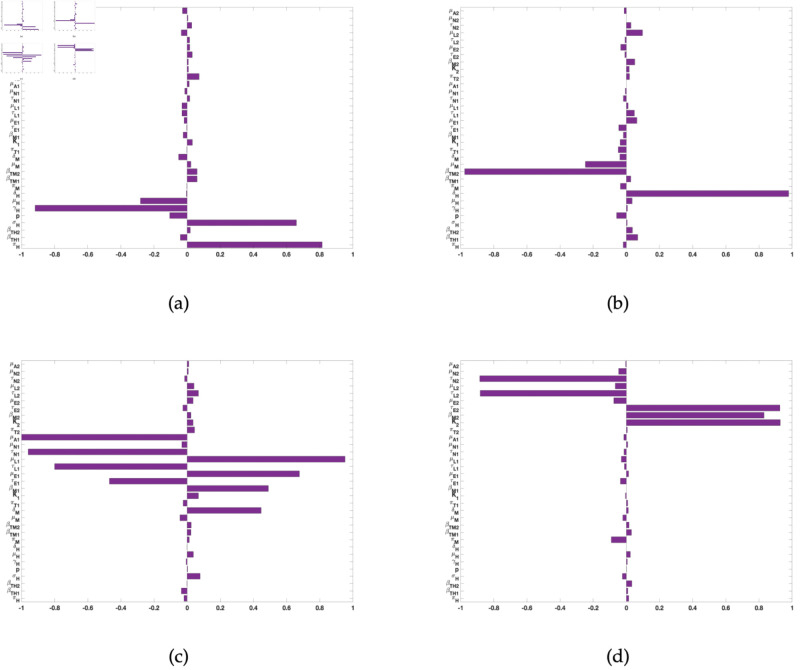
PRCC values for the tularaemia model (1), using as response functions: (a) The infected humans (*I*_*H*_); (b) The infected rodents (*I*_*M*_); (c) The sum of infected Dermacentor variabilis ticks in all life stages ($I_{L1}+I_{N1}+I_{A1}$); and (d) The sum of infected Amblyomma americanum ticks in all life stages ($I_{L2}+I_{N2}+I_{A2}$). Using parameter values in Table 2 and ranges that are ±20% from the baseline values.

For the other response functions (see Fig 3), the most significant parameters related to the infected humans, for instance are humans birth and death rates ($\pi_{H},\;\mu_{H})$, disease progression rate ($\sigma_{H}$), and the recovery rate ($\gamma_{H}$). For the infected rodent response function, the significant parameters are rodent transmission probability ($\beta_{TM2}$) to *A. americanum* and human disease induced death rate ($\delta_{H}$). For the response function related to the sum of infected *D. variabilis* ticks in all life stages ($I_{L1}+I_{N1}+I_{A1}$), the significant parameters are the mortality rate ($\mu_{A1}$) of adult *D. variablis* ticks, the maturation rate ($\tau_{N1}$) of *D. variablis* nymphs, the mortality ($\mu_{L1}$) and maturation ($\tau_{L1}$) rates of larvae, eggs in-viability ($\mu_{E1}$) and maturation ($\tau_{E1}$) rates for *D. variablis*, and transmission probability ($\beta_{M1}$) of *D. variablis* to rodents infection; finally for this response function is the rodent disease-induced death rate ($\delta_{M}$). Lastly, for the sum of infected *A. americanum* ticks ($I_{L2}\;+\;I_{N2}\;+\;I_{A2}$) response function, the significant parameters are the carrying capacity (*K*_2_) of *A. americanum*, the transmission probability $(\beta_{M2}$) of *A. americanum* to-rodent, the eggs maturation ($\tau_{E2}$) rate, the larvae maturation rate ($\tau_{L2}$), and the nymphs maturation ($\tau_{N2}$) rate.

The PRCC index values of some of these parameters have positive signs while others have negative signs. The positive signs means that any increase in these parameters will lead to an increase in all the response functions. While the negative signs means that an increase in the parameters will lead to a decrease in the response functions. Hence, these parameters would be useful targets during mitigation efforts. Therefore, control strategies which targets these parameters with significant PRCC values will give the greatest impact on the model response functions.

#### 2.4.2 Global sensitivity analysis for tularaemia model (2).

For the sensitivity analysis for tularaemia model (2), we included the number of burns (pulses) and the time between the burns (*τ*) and drew from Poisson distributions for these parameters. We then used as response functions the sum of infected *Dermacentor variabilis* ticks in all life stages ($I_{L1}+I_{N1}+I_{A1}$) and, the sum of infected *Amblyomma americanum* ticks in all life stages ($I_{L2}+I_{N2}+I_{A2}$).

**Fig 4:**
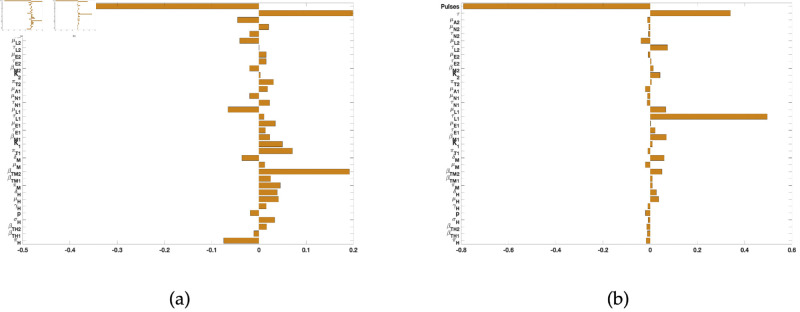
PRCC values for the tularaemia model (1), using as response functions: (a) The sum of infected Dermacentor variabilis ticks at all life stages ($I_{L1}+I_{N1}+I_{A1}$); and (b) The sum of infected Amblyomma americanum ticks at all life stages ($I_{L2}+I_{N2}+I_{A2}$). Using parameter values in Table 2 and ranges that are ±20% from the baseline values.

For the two response functions, the number of burns (pulses) and the time between the burns (*τ*) were the most significant parameters with negative and positive PRCC values followed by $\tau_{L1}$ and $\beta_{TM2}$. The sign on the pulses (negative) implies a negative impact on two ticks species. The sign on *τ* on the other hand is positive pointing to a positive impact on the ticks (Fig 4). In the next section below we will explore the impact of these two parameters on our model.

Thus, as the frequency of the burn increases few ticks will remain. That is, as the time between the burn increases more ticks will be found (Fig 4).

## 3 Results

### 3.1 Effect of prescribed fire

Our goal in this research work is to investigate the differential effect of fire on the two tick species and the prevalence of tularemia disease on the two host populations (humans and rodents). To address our research goal, we simulate the tularemia model (2) with prescribed and compare the outcome to the tularemia model (1) without prescribe fire. We focus on infected humans, rodents, and the nymph populations of the two tick species.

**Fig 5:**
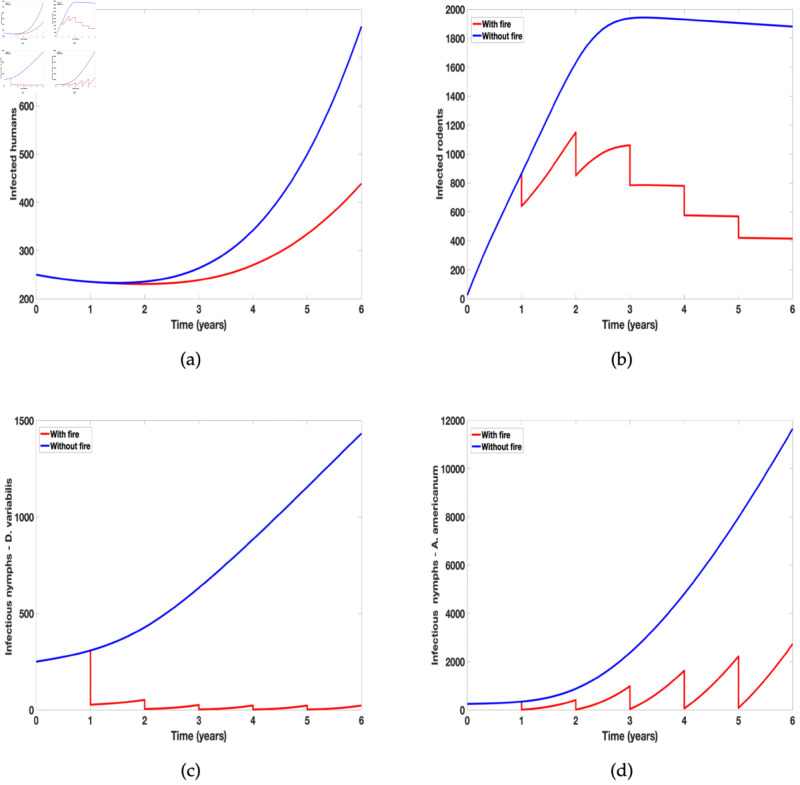
Simulation of tularaemia model (1) showing the effect of prescribed fire on: (a) Infected humans (*I*_*H*_); (b) Infected rodents (*I*_*M*_); (c) Infected Dermacentor variabilis (*I*_*N*1_); and (d) Infected Amblyomma americanum (*I*_*N*2_). Using parameter values in Table 2.

In Fig 5 we observed a substantial difference between the number of humans and rodents infected with tularemia when prescribed fire is implemented and when it is absent (see Fig 5(a) and 5(b)). Although we have the same number of infected humans and rodents in the first year before the application of fire but once prescribed fire in implemented we see a reduction in infected humans and rodent population. We observed similar dynamics in both infected *D. variabilis* and *A. americanum* ticks but the population of *A. americanum* rebound quicker than those of *D. variablis*. Thus showing the differential effect of prescribed fire on the two tick species.

### 3.2 Differential effect of fire on infected new cases

**Fig 6:**
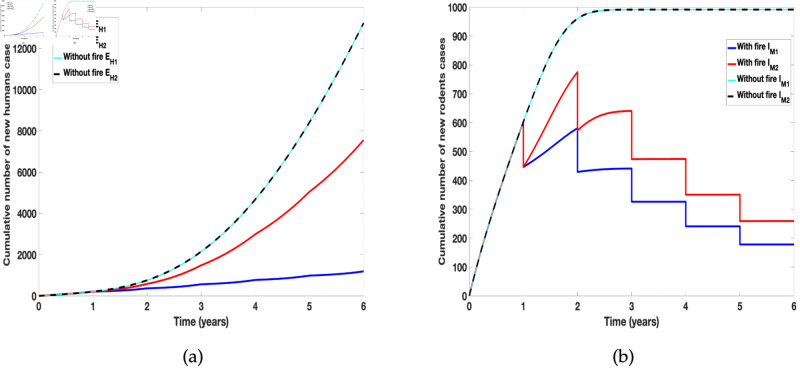
Simulation of tularaemia models (1) and (2) showing differential effect of prescribed fire on: (a) Cumulative number of new infected human cases; (b) Cumulative number of new infected rodent cases. Using parameter values in Table 2 and assuming the ticks related parameter values for the two tick species are the same.

Next, we consider the differential effect of prescribed fire on cumulative humans and rodents infected new cases. To account for the cumulative number of new cases, we consider for humans the transition to the exposed class (*E*_*H*_) from the susceptible class (*S*_*H*_) due to the individual tick species. Similarly for the rodents, we consider the transition into the infected class (*I*_*M*_) due to the individual ticks (see the force of infection for humans in models (1) and (2)). We then assumed all the ticks related parameter values are the same except for the proportion reduced by fire. Fig 6 reveals the outcome of the simulation clearly showing the differential effect of fire on the cumulative number of new infected human and rodent cases. Furthermore, we observed the difference in the cumulative number of new cases due to the individual ticks, with *A. americanum* producing more infections than *D. variablis*, since many of them are remaining after each burn. Observe that in the absence of fire, the number of new cases from the infectious ticks of the two species are the same.

**Fig 7:**
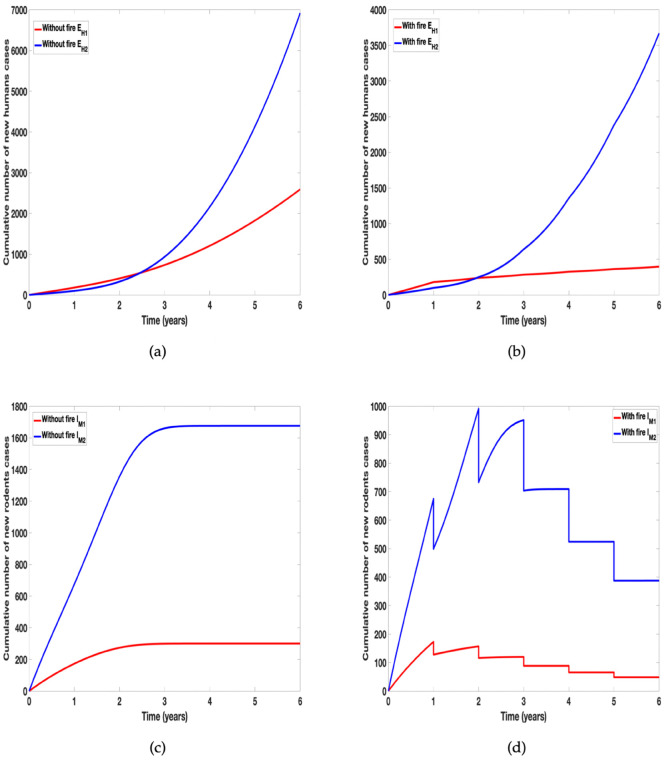
Simulation of tularaemia models (1) and (2) showing differential effect of prescribed fire on: (a) Cumulative number new infected humans cases in the absence of fire; (b) Cumulative number of new infected humans cases in the presence of fire; (c) Cumulative number of new infected rodents cases in the absence of fire; and (d) Cumulative number of new infected rodents cases in the presence of fire. Using parameter values in Table 2 with different tick related parameter values for the two tick species.

Next we simulate the models to show the differential effect of prescribed fire on the incidence of the disease due to individual tick species when the tick related parameter values for the two tick species are different. Here the parameter values in Table 2 are used for the simulations and we can further see the differential effect of prescribed fire on the incidence of the disease, see Fig 7.

### 3.3 Frequency and time between burn

**Fig 8:**
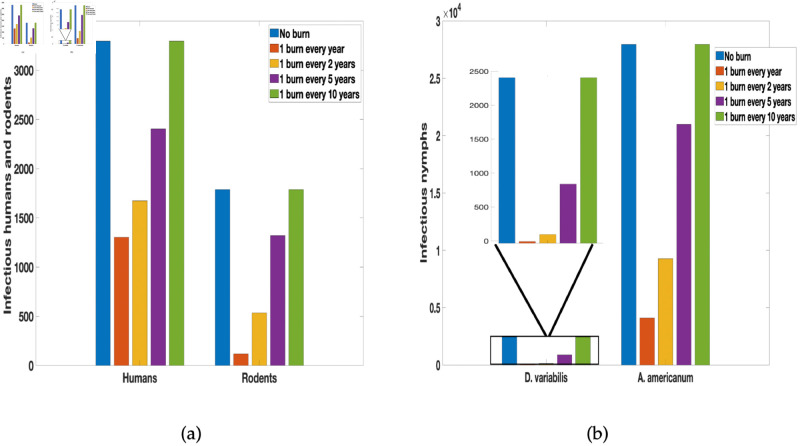
Simulation of tularaemia model (2) with prescribed fire with different time between burn using the baseline prescribed fire reduction parameters obtained from [2, 76] and the rest of the parameter values in Table 2.

Next, we consider the time between each burn and explore the effect of the frequency of the burn over ten years. We consider different burning regimes, for instance, we burn annually for a period of ten years, once every other year, once every five years, and once every ten years. We found in Fig 8 more infected humans and rodents as duration between burns increases. Similar increase in the number of infectious nymph populations is observed as the duration between each burn increases. However, the frequently prescribed fire is implemented the fewer the infected populations (humans, rodents, and ticks).

### 3.4 Burn environments

Next, we consider the effect of prescribed fire in different burn environments on disease transmission in humans and rodents. We use the 2010 burn data from Gleim *et al*. [43] in two burn environments namely the unburned area surrounded by burned area (UBB) and burned area surrounded by unburned area (BUB).

Gleim *et al*. conducted their study in southwestern Georgia and northwestern Florida, environments dominated by pine and mixed pine forests and agriculture. They selected 21 sites (two privately owned, 8 state-owned, and 11 owned by the J.W. Jones Ecological Research Center) based on the long history of prescribed burning or no burning. The burnt sites had at least 10 years of regular prescribed burns and the unburned sites had no history of prescribed burning for more than 10 years. Each plot was sampled monthly for ticks for 24 months (January 2010-December 2011) using 1 m×1 m flags made of flannel cloth. Flagging across the sites was conducted only when the temperature was above $7.2^{\text{o}}$C and after 10 AM when there was no dew or moisture from precipitation (that is, the vegetation was dry). Furthermore, the research team did not flag the sites during inclement weather such as rain, snow, or excessive wind. The sites were flagged for a minimum of one hour per site per month. Gleim *et al*. did not collect data for the rodent population, so we used our baseline data obtained from [2, 76] for the rodent population, see Table 3.

**Table 3:** The 2010 burn data from Gleim *et al*. [43] and simulation outputs in unburned area surrounded by burned area (UBB) and burned area surrounded by unburned area (BUB) using the fire reduction proportions $\nu_{Li},\;\nu_{Ni},\;\nu_{Ai},\;\nu_{M},$ i=1,2 estimated from data and simulated values for UBB: $\nu_{A1}=0.992,\;\nu_{L2}=0.9$, BUB: $\nu_{A1}=0.99,$ $\nu_{L2}=0.942$.

D. variabilis	Treatment	Adult	Nymph	Larvae
Data	UBB	7	0	0
	BUB	9	0	0
Simulation	UBB	7	0	0
	BUB	9	0	0
*A. americanum*	Treatment	Adult	Nymph	Larvae
Data	UBB	5	24	2354
	BUB	4	2	1362
Simulation	UBB	2	35	2344
	BUB	3	5	1360

Given the data in Table 3, we determine the values of the parameters $\nu_{Li},\;\nu_{Ni}$, and $\nu_{Ai}$, i=1,2 by dividing the numbers of ticks collected in each age group by the total number collected and then subtracting the proportion obtained from 1 to obtain the proportion reduced by fire. However, in the case of *D. Variabilis*, the proportion of adult ticks collected after the burn in the two environment are close to one or are one. To determine the values for $\nu_{Ai}$, we gradually adjusted the values of $\nu_{Ai}$ and simulated the model till the number of ticks are close to the collected data. Given these proportions, we then simulated the tularemia model (2) for these two burn environments for a year and compare the outcome to the data, see Table 3. The table shows the simulation results closely match the data especially for D. variabilis in both burn environments.

With these estimated proportions $\nu_{Li},\;\nu_{Ni}$, and $\nu_{Ai}$, we simulated the model for a period of five years to determine the effect of prescribed fire in the different burn environments on disease transmission in humans and rodents. The different burn environments are the no burn environment, the UBB, BUB, and the baseline burn environments using proportions obtained in Sect 2.3.1 above.

**Fig 9:**
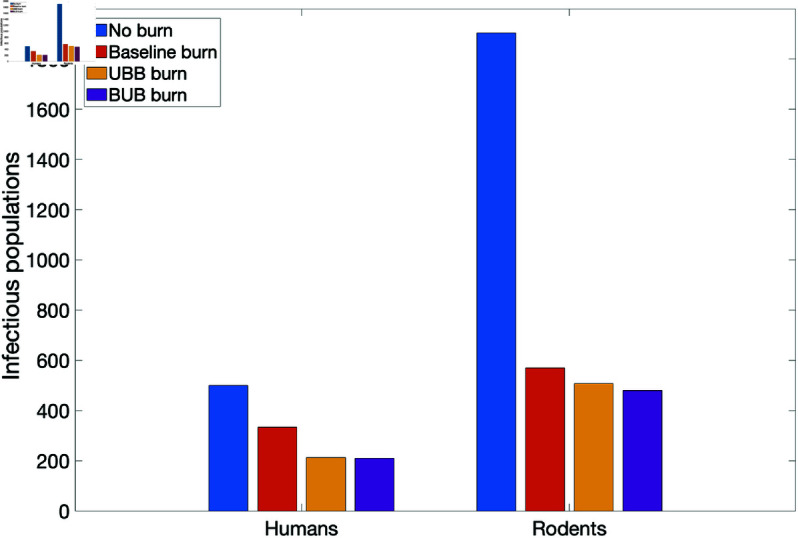
Simulation of tularaemia model (2) with prescribed fire in different environments for infected humans and rodents. The baseline prescribed fire reduction parameters were obtained from [2, 76]. The reduction parameters in UBB and BUB were estimated using Gleim *et al*. 2010 data [43], the rest of the parameter values are in Table 2.

In Fig 9, we observed that the no burn environment led to the most infected humans and rodents, while fewer infected was observed in UBB and BUB environments. However, a relatively more infected humans and rodents are in UBB compare to BUB, see Table 4 for the resulting number of infectious nymphs for the two tick species in each of the environments at the end of the simulation period. As expected, there are more *A. americanum* than *D. variabilis*.

**Table 4:** Simulation results of the tularaemia model (2) at the end of five years with prescribed fire in different environments using estimated fire reduction proportions $\nu_{Li},\;\nu_{Ni},\;\nu_{Ai},\;\nu_{M},$ i=1,2 estimated from data and simulated values for UBB: $\nu_{A1}=0.992,\;\nu_{L2}=0.9$, BUB: $\nu_{A1}=0.99,$ $\nu_{L2}=0.942$.

Burn environment	Humans	Rodents	*D. variabilis*	*A. americanum*
No burn	499.85	$1.91\times 10^{3}$	1.16$\times 10^{3}$	7.98$\times 10^{3}$
Baseline burn	334.22	568.82	22.52	2.22$\times 10^{3}$
UBB burn	212.52	507.42	7.76	144.03
BUB	210.48	480.12	7.28	114.65

## 4 Discussion and conclusion

### Discussion

In this paper we developed and analyzed a mathematical model for a tick-borne tularemia disease transmission dynamics in humans, rodents and two ticks species (*Dermacentor variabilis* and *Amblyomma americanum*) using the natural history of infection of the disease; the model incorporates prescribed fire. Tularemia is a zoonotic disease caused by *Francisella tularensis* bacteria [4, 6, 10, 11], a gram-negative coccobacillus-shaped bacterium; There are four subspecies of *F. tularensis* namely *tularensis* (type A), *holarctica* (type B), *novicida* and *mediasiatica* [4, 11]. The most virulent subspecies are *tularensis* and *holarctica* [6, 11, 15–17]. The subspecies *tularensis* and *holarctica* are the major etiological agents of tularemia in humans [4]. There are multiple transmission routes of the infection to humans such as consumption of contaminated food or water, handling of infected animals or bites from haematophagous arthropod vectors (such as ticks, deer flies, or mosquitoes) [6, 19, 20]; other routes include contact with aquatic environment, and inhalation of aerosols [4, 21–23]. In this study we focus on transmission via the bites of ticks.

The goal of the study is to explore the differential effect of fire *via* prescribed burn on the two ticks species and how that affects the prevalence of the disease in humans and rodents. Prescribed fire is a common and essential land management technique used in ecosystems that depend on fire, such as grasslands, open pine forests, and wetlands [39, 40]. Prescribed fire was used often used by the native Americans long before the era of fire suppression. These practises are been revived by several native tribes across the United States [85, 86]. These cultural fires help to repair degraded soil and increase biodiversity while reducing the leave litters and overgrowth that contributes to wild fires [85–87]. The native plants, which have adapted to fire are not harm by these cultural burns, in fact many of these plants need fire for their seeds to germinate [85].

Quantitative analysis of the tularemia model (1) without fire indicate that the model has a disease-free equilibrium that is locally asymptotically stable when the reproduction number is less than one. The global sensitivity analysis of this model using LHS/PRCC method was carried out to determine the parameter with the most influence on several response functions like the reproduction number (${ℛ}_{0}$), the infected humans (*I*_*H*_), the infected rodents (*I*_*M*_), the sum of infected *D. variabilis* ticks in all life stages ($I_{L1}\;+\;I_{N1}\;+\;I_{A1}$), and the sum of infected *A. americanum* ticks in all life stages ($I_{L2}+I_{N2}+I_{A2}$). To implement the analysis, we used parameter values obtained from literature in most cases as well as assumed some where we could not find their values. Identification of these parameters are valuable for targeting interventions and perhaps for gathering data for other analyzes.

The parameters with the most significant impacts on the response functions are rodent birth rate ($\pi_{M}$), rodent transmission probability ($\beta_{TM2}$) to *A. americanum*, rodent death ($\mu_{M}$) and disease induced mortality ($\delta_{M}$) rates, *A. americanum* carrying capacity (*K*_2_), transmission probability ($\beta_{M2}$) of tularemia to rodents from *A. americanum*, and the maturation rate ($\tau_{E2}$) of *A. americanum* larvae and its adult death rate ($\mu_{A2})$ (see Figs 2 and 3). Others include humans birth and death rates ($\pi_{H},\;\mu_{H})$, disease progression rate ($\sigma_{H}$), and the recovery rate ($\gamma_{H}$), human disease induced death rate ($\delta_{H}$). The significant parameters also include the mortality rate ($\mu_{A1}$) of adult *D. variablis* ticks, the maturation rate ($\tau_{N1}$) of *D. variablis* nymphs, the mortality ($\mu_{L1}$) and maturation ($\tau_{L1}$) rates of larvae, eggs in-viability ($\mu_{E1}$) and maturation ($\tau_{E1}$) rates for *D. variablis*, and transmission probability ( $\beta_{M1}$) of *D. variablis* to rodents infection, rodent disease-induced death rate ($\delta_{M}$), the eggs maturation ($\tau_{E2}$) rate, the larvae maturation rate ($\tau_{L2}$), and the nymphs maturation ($\tau_{N2}$) rate.

The sensitivity analysis for tularemia model (2) with prescribed fire was also carried out using as response functions the sum of infected *D. variablis* ticks ($I_{L1}\;+\;I_{N1}\;+\;I_{A1}$) and the sum of infected *A. americanum* ($I_{L2}\;+\;I_{N2}\;+\;I_{A2}$) ticks at all life stages as the model without fire; the number of burns (pulses) and the time between the burns (*τ*) were drawn from Poisson distributions. For the two response functions, the number of burns (pulses) and the time between the burns (*τ*) were the most significant parameters with PRCC values having negative and positive signs meaning negative and positive impact on the two ticks species. Thus, as the frequency of prescribed fire increases fewer ticks will remain or will be found. Furthermore, as the time between the burn increases more ticks will be found since their population would have increased in the time before the implementation of the next burn which will lead to reduction of their population. This result aligns with the outcome obtained by Guo and Agusto [2], and Fulk *et al*. [46, 47].

Arthropods have complex reactions to fire [88]; their responses to fire depend on the intensity and severity of the fire. Fire can lead to abundance or reduction in their population and composition, so much so that only certain arthropods will be able to live in habitats that experience frequent fires especially wildfires [88]. In Figs 6 and 7 we examine the differential effect of fire on the incidence of tularemia due to the two species using data collected by Gleim *et al*. [43, 89]. The data showed more *A. americanum* than *D. variablis* were collected after the different burn regimes. This abundance was evident in the cumulative number of new human and rodent infection due to the tick species. To clearly show the differential effect of fire, we assumed the same parameter values for the two species except for the proportion reduced due to fire. As expected *A. americanum* which was reduced the least produced more infection, see Fig 6. In Fig 7, we relaxed this assumption and simulated the model using the parameters in Table 2; this figure further emphasis the result observed in Fig 6 that *A. americanum* led to more infection than *D. variablis* due to its abundance after the burn.

The numerical simulations of the tularaemia model (2) depicted in Fig 5, offer valuable insights into the dynamic interplay between prescribed fire and the spread of tularemia. The observed decrease in the number of infected, particularly in tick species *D. variablis* and *A. americanum*, underscores the potential efficacy of prescribed fire in mitigating the prevalence of the disease. This result aligns with the results obtained by Guo and Agusto [2] which showed reduction in Lyme disease with the use of prescribed fire. Guo and Agusto [2] further showed that high intensity burn reduces more ticks than low intensity burn. This current study further aligns with study by Fulk *et al*. [46] which showed reduction in the prevalence of Lyme disease in different spatial setting (grassland or wooded area) with the application of prescribed burn. Similar results was also obtained in the study by Fulk and Agusto [47] on the effects of rising temperature and prescribed fire on *A. americanum* with ehrlichiosis. The study found that with significant increase in temperature as high a 2 °C or 4 °C, implementing prescribed burning can still lead to reduction in the prevalence of infectious questing nymphs and adults with ehrlichiosis.

Using the results from the sensitivity analysis we explore in Fig 8 the frequency and time between burns. The parameters used for the proportion reduced as a result of fire was obtained from [2, 76]. Considering the results from Fig 8, there is a considerable reduction in infected humans and rodents as well as the infectious nymphs generally as a result of the burn. However, the frequency of the burn plays a significant role in ensuring whether there is a significant reduction or not. As can be deduced from the figure, one burn every year ensures a significant reduction in the infected (humans, rodents, and ticks). The simulation results emphasize the importance of well-timed fire applications in controlling tularemia. This result confirms the results from Guo and Agusto [2] which explore the effect of fire frequency and the time between burns. As identified in the study, the duration between burns has a more significant effect on ticks with higher-intensity fires than with lower-intensity fires. However, fire intensity appears to have a larger influence on tick reduction than the duration of the burns, as burning fewer times at a higher intensity is more effective than burning more times at a lower intensity.

The exploration of the different burn plots in Fig 9 reveals the infection patterns in the two environments (UBB and BUB). The number of infected humans and *D. variabilis* population in these environments are relatively the same. Although the larvae and nymph populations for *D. variablis* were zero in data shown in Table 3, some adults survived the burn and remained. Generally, surviving adults can lead to new population that at times can grow rapidly to the pre-burn levels [90]. This of course depend on factors like tick species, habitat types, environmental climate, and frequency of burns [2, 46, 90]. This explains the low numbers of infectious *D. variablis* nymphs in Table 4 and the numbers observed in the simulation trajectories (not shown here). Furthermore, a lot of larvae stage *A. americanum* remained in both environments after the prescribed burn, indicating the differential effect of fire and these ticks high level of tolerance to fire (see Gleim *et al*. [43] and Table 3). Infection was high in the two burn environments for *A. americanum* and the rodent populations. This is likely due to the fact that *A. americanum* at the larvae and nymphal stages are aggressive biters that readily seeks out hosts like the rodents [91]. However, frequent and long-term prescribed fire will significantly reduce their abundance over time regardless of sites such as UBB and BUB [43, 89].

Hence, the results of this study suggests that the spatial arrangement of burned and unburned areas regarding tularemia infection in humans and *D. variabilis* does not matter while the type of the environment matters for *A. americanum* and the rodents since there were more infected ticks and rodents in UBB compare to BUB, see Tables 3 and 4. Furthermore, the overall impact of prescribed fire on *D. variabilis* is significant unlike the effect on *A. americanum*, thus indicating the differential effect of fire on the tick spices.

### Conclusion

To conclude, in this study we developed a deterministic model of ordinary and impulsive differential equations to gain insight in the differential effect of prescribed fire on *Dermacentor variabilis* and *Amblyomma americanum* ticks and the prevalence of tularemina. We found that prescribed fire can differentially reduce the number of the two ticks with *D. variablis* been reduced the most compare to *A. americanum*. We summarize the other results as follows:

(i) The results of the sensitivity analyzes using as response or output functions the reproduction number (${ℛ}_{0}$), the infected humans (*I*_*H*_), the infected rodents (*I*_*M*_), the sum of infected *D. variablis* ticks at all life stages ($I_{L1}\;+\;I_{N1}\;+\;I_{A1}$), and the sum of infected *A. americanum* ticks at all life stages ($I_{L2}\;+\;I_{N2}\;+\;I_{A2}$) to identify the parameters with the most impact on these functions in no particular order are: the most significant parameters related infected humans are the birth, death, and disease induced death rates ($\pi_{H},\;\mu_{H}$, $\delta_{H}$), human disease progression rate ($\sigma_{H}$), and the recovery rate ($\gamma_{H}$); the most significant parameters related to infected rodents are rodent birth, death, and disease induced death rates ($\pi_{M}$, $\mu_{M}$, $\delta_{M}$), rodent transmission probability ($\beta_{TM2}$) to *A. americanum*. The significant parameters related *D. variablis* are eggs maturation ($\tau_{E1}$) and in-viability ($\mu_{E1}$) rates, the larvae maturation ($\tau_{L1}$) and mortality ($\mu_{L1}$) rates, the nymphs maturation rate ($\tau_{N1}$), and the adult mortality rate ($\mu_{A1}$). The significant parameters related to *A. americanum* are its carrying capacity (*K*_2_), the eggs maturation ($\tau_{E2}$) rate, the larvae maturation rate ($\tau_{L2}$), and the nymphs maturation ($\tau_{N2}$) rate, its adult death rate ($\mu_{A2})$, and the transmission probability ($\beta_{M2}$) to rodents.
(ii) Prescribed fire can reduce the number of ticks leading to a reduction in the number of tularemia infected humans, rodents and ticks.
(iii) *A. americanum* produced more tulareamia infection in humans and rodents than *D. variablis* due to the differential effect of prescribed fire which leaves more *A. americanum* than *D. variablis* after a burn.
(iv) As time between burn increases, more infected humans, rodents, and ticks increases. Frequent burning reduces the number of ticks and therefore infections.
(v) The spatial arrangement of UBB and BUB plots may not matters for humans and *D. variabilis* unlike *A. americanum* and the rodents which had more infected in UBB compare to BUB.

In this study, we have used mathematical models to investigate the differential effect of prescribed burn on two tick species namely *D. variablis* and *A. americanum* and explored the effectiveness of prescribed fire in managing tick populations and lowering the prevalence of tularemia disease in the population (humans, rodents, and ticks). Our study is not without some limitations; the biggest drawbacks was the availability of data. For instance, there are no rodent data in the UBB and BUB environment. Despite this drawback, we have used the limited available data and we understand it might affect our results but we have confidence in our conclusions given the results from the sensitivity analysis we carried out and the validation of the data shown in Table 3 via simulation approaches.

Occhibove *et al*. [58] found that the degree of vector generalism affected pathogen transmission with different dilution outcomes. *A. americanum* is known as an aggressive and generalist hematophagous tick species [92–94]. In future studies, we will explore the differential effect of prescribed fire on the dilution or amplification tendencies of this tick species on the transmission dynamics of tick-borne diseases. Furthermore, we will equally explore other ecological implications of our findings, by considering factors such as habitat fragmentation and species interactions in the presence of prescribed fire. Lastly, we will explore ways to apply these outcomes to other tick management and control strategies.

## Supporting information

S1 Appendix Proof of Lemma 1. (PDF)

S2 Appendix Proof of Lemma 2. (PDF)
