# Supplementary material for: Modeling the differential effect of prescribed fire on multi-vector tick-borne tularemia disease
Source: PLoS One. 2025 Aug 11;20(8):e0329465. doi: 10.1371/journal.pone.0329465 (PMC12338822; doi:10.1371/journal.pone.0329465)
Supplement: S1 Appendix — (PDF) [file pone.0329465.s001.pdf]

Supporting information

S1 Appendix Proof of Lemma 1.

Lemma 1: Let the initial data $F(0) \geq 0$, where $F(t) = (S_H(t), E_H(t), A_H(t), I_H(t), R_H(t), S_M(t), I_M(t), S_{Ei}(t), S_{Li}(t), I_{Li}(t), S_{Ni}(t), I_{Ni}(t), S_{Ai}(t), I_{Ai}(t))$, where $i = 1, 2$ ticks. Then the solutions $F(t)$ of the tularemia model (1) are non-negative for all $t > 0$. Furthermore

$$\limsup_{t\to\infty} N_H(t) \leq \frac{\pi_H}{\mu_H}, \quad \limsup_{t\to\infty} N_M(t) \leq \frac{\pi_M}{\mu_M}, \quad \textbf{and} \quad \limsup_{t\to\infty} N_{Ti}(t) \leq \frac{\pi_{Ti}}{\mu_{Ti}} \limsup_{t\to\infty}.$$

where

$$N_H(t) = S_H(t), E_H(t), A_H(t), I_H(t), R_H(t), \qquad N_M(t) = S_M(t), I_M(t),$$

and

$$N_{Ti}(t) = S_{Ei}(t), S_{Li}(t), I_{Li}(t), S_{Ni}(t), I_{Ni}(t), S_{Ai}(t), I_{Ai}(t).$$

Proof. Let $t_1 = \sup\{t > 0 : F(t) > 0 \in [0, t]\}$. Thus, $t_1 > 0$. It follows from the first equation of the system (1), that

$$\frac{dS_H}{dt} = \pi_H - \lambda_H S_H - \mu_H S_H D$$

which can be re-written as

$$\frac{d}{dt}\left\{S_H(t)\ exp\left(\int_0^{t_1} \lambda_H(\zeta)d\zeta + \mu_H t\right)\right\} \;=\; \pi_H\ exp\left(\int_0^{t_1} \lambda_H(\zeta)d\zeta + \mu_H t\right),$$

Hence,

$$S_H(t_1)\ exp\left(\int_0^{t_1} \lambda_H(\zeta)d\zeta + \mu_H t_1\right) - S_H(0) \quad = \quad \int_0^{t_1} \pi_H\ exp\left(\int_0^{p} \lambda_H(\zeta)d\zeta + \mu_H p\right)dp$$

so that,

$$\begin{aligned} S_H(t_1) \quad &= \quad S_H(0)\ exp\left[-\left(\int_0^{t_1} \lambda_H(\zeta)d\zeta + \mu_H t_1\right)\right] + exp\left[-\left(\int_0^{t_1} \lambda_H(\zeta)d\zeta + \mu_H t_1\right)\right] \\ &\times \quad \int_0^{t_1} \pi_H\ exp\left[\left(\int_0^{p} \lambda_H(\zeta)d\zeta + \mu_H p\right)\right]dp \\ &> \quad 0. \end{aligned}$$

Similarly, it can be shown that $F > 0$ for all $t > 0$.

For the second part of the proof, note that $0 < S_H(0) \leq N_H(t), 0 \leq E_H(0) \leq N_H(t), 0 \leq A_H(0) \leq N_H(t), 0 < I_H(0) \leq N_H(t), 0 \leq R_H(0) \leq N_H(t), 0 < S_M(0) \leq N_M(t), 0 \leq I_M(0) \leq N_M(t), 0 < S_{Ei}(0) \leq N_{Ti}(t), 0 < S_{Li}(0) \leq N_{Ti}(t), 0 \leq I_{Li}(0) \leq N_{Ti}(t), 0 < S_{Ni}(0) \leq N_{Ti}(t), 0 \leq I_{Ni}(0) \leq N_{Ti}(t), 0 < S_{Ai}(0) \leq N_{Ti}(t), 0 \leq I_{Ai}(0) \leq N_{Ti}(t)$, where $i = 1, 2$ ticks.

Adding the human, rodent, and tick component of the tularemia model (1) gives

$$\frac{dN_H(t)}{dt} = \pi_H - \mu_H N_H(t) - \delta_H I_H(t), \tag{S.1}$$

$$\frac{dN_M(t)}{dt} = \pi_M - \mu_M N_M(t) - \delta_M I_M(t), \tag{S.2}$$

$$\frac{dN_{Ti}(t)}{dt} = \pi_{Ti} - \mu_{Ti} N_{Ti}.$$

Hence,

$$\limsup_{t\to\infty} N_H(t) \le \frac{\pi_H}{\mu_H}, \quad \limsup_{t\to\infty} N_M(t) \le \frac{\pi_M}{\mu_M}, \quad \text{and} \quad N_{Ti}(t) = \frac{\pi_{Ti}}{\mu_{Ti}} \quad \limsup_{t\to\infty}.$$

as required. □
