# Supplementary material for: Modeling the differential effect of prescribed fire on multi-vector tick-borne tularemia disease
Source: PLoS One. 2025 Aug 11;20(8):e0329465. doi: 10.1371/journal.pone.0329465 (PMC12338822; doi:10.1371/journal.pone.0329465)
Supplement: S2 Appendix — (PDF) [file pone.0329465.s002.pdf]

S2 Appendix Proof of Lemma 2.

Lemma 2: The region $\Omega = \Omega_H \cup \Omega_M \cup \Omega_{T1} \cup \Omega_{T2} \subset \mathbb{R}^5_+ \times \mathbb{R}^2_+ \times \mathbb{R}^7_+ \times \mathbb{R}^7_+$ is positively-invariant for the tularemia model (1)-(2) with non-negative initial conditions in $\mathbb{R}^{19}_+$.

Proof. It follows from the sum of the first five equations of the tularemia model (1) that

$$\frac{dN_H(t)}{dt} = \pi_H - \mu_H N_H(t) - \delta_H I_H(t), \tag{S.3}$$

$$\frac{dN_H(t)}{dt} \leq \pi_H - \mu_H N_H(t).$$

Hence, $\dfrac{dN_H(t)}{dt} \leq 0$, if $N_H(0) \geq \dfrac{\pi_H}{\mu_H}$. Thus,

$$N_H(t) \leq N_H(0)e^{-\mu_H t} + \frac{\pi_H}{\mu_H}(1 - e^{-\mu_H t}). \tag{S.4}$$

In particular, if $N_H(0) \leq \dfrac{\pi_H}{\mu_H}$, then $N_H(t) \leq \dfrac{\pi_H}{\mu_H}$.

Next, summing the equations for the rodents, the tularemia model (1) gives the following

$$\frac{dN_M(t)}{dt} = \pi_M - \mu_M N_M(t) - \delta_M I_M(t), \tag{S.5}$$

$$\frac{dN_M(t)}{dt} \leq \pi_M - \mu_M N_M(t).$$

Hence, $\dfrac{dN_M(t)}{dt} \leq 0$, if $N_M(0) \geq \dfrac{\pi_M}{\mu_M}$. Thus,

$$N_M(t) \leq N_H(0)e^{-\mu_M t} + \frac{\pi_M}{\mu_M}(1 - e^{-\mu_M t}). \tag{S.6}$$

In particular, if $N_M(0) \leq \dfrac{\pi_M}{\mu_M}$, then $N_M(t) \leq \dfrac{\pi_M}{\mu_M}$.

Lastly, the ticks equations of the tularemia model (1) gives the following after summing the equations representing the eggs, larvae, nymphs, and adult stages for each of the ticks population

$$\frac{dN_{Ti}(t)}{dt} = \pi_{Ti} - \mu_{Ti} N_{Ti}, \tag{S.7}$$

where $\mu_{Ti} = \min\{\mu_{Ei}, \mu_{Li}, \mu_{Ni}, \mu_{Ai}\}$ and $i = 1, 2$ ticks. Thus,

$$N_{Ti}(t) = \frac{\pi_{Ti}}{\mu_{Ti}} + \left(N_{Ti}(0) - \frac{\pi_{Ti}}{\mu_{Ti}}\right)e^{-\mu_{Ti} t}. \tag{S.8}$$

Equations (S.3), (S.5), and (S.7) implies that $N_H(t)$, and $N_{Ti}(t)$ are bounded and all solutions starting in the region Ω remain in Ω. Thus, the region is positively-invariant and hence, the region Ω attracts all solutions in $\mathbb{R}^{19}_+$.

$\square$
